# PolarTIL programming reveals an essential role for TGF-β1 in antitumor memory in human cutaneous malignancies

**DOI:** 10.21203/rs.3.rs-11309475/v1

**Published:** 2026-10-30

**Authors:** Megan Wyatt, Rachel Mason, James Carmouche, Ayana Ruffin, Megen Wittling, Anna Cole, Aubrey Smith, Guillermo Rangel Rivera, Michael Ware, Michael Lowe, Keith Delman, Nathaniel Wilson, Gregory B. Lesinski, Chrystal Paulos

**Affiliations:** Winship Cancer Institure of Emory University; Winship Cancer Institute, Emory University; Winship Cancer Institute, Emory University; Emory University; Emory University; Winship Cancer Institute, Emory University; Winship Cancer Institute, Emory University; Winship Cancer Institute, Emory University; Winship Cancer Institute, Emory University; Emory University School of Medicine; Emory University; Winship Cancer Institute, Emory University; Emory University; Winship Cancer Institute, Emory University

## Abstract

Tumor-infiltrating lymphocyte (TIL) therapy can induce tumor regression in solid malignancies, yet durable responses remain limited, highlighting the need for more effective cell products. Here, we generated a novel TIL product, PolarTIL, by polarizing murine and human TIL from cutaneous malignancies ex vivo with a defined Type-17 cytokine cocktail. Compared with conventional IL-2 culture, PolarTIL produced greater cell yields and a distinct cellular state marked by reduced CD4^+^ regulatory T cells, enrichment of CD103^+^ populations, and superior antitumor activity *in vitro* and *in vivo*. Unexpectedly, TGF-β1 emerged as a critical regulator of PolarTIL function. Although TGF-β is commonly targeted for inhibition in cancer immunotherapy, its removal during PolarTIL generation completely abolished therapeutic efficacy *in vivo*. Together, these findings reveal an unrecognized requirement for TGF-β1 in programming effective TIL responses and establish PolarTIL as a promising next-generation cellular therapy for solid tumors.

## Introduction

Adoptive transfer of tumor-infiltrating lymphocytes (TIL) can mediate clinical responses in patients with aggressive solid malignancies, including metastatic melanoma, cholangiocarcinoma, and lung cancers^1-3^. Although FDA-approved for metastatic melanoma, some studies suggest that prior immunotherapy may impact the subsequent function of TIL therapy^4,5^, exposing fundamental limitations. Conventional *ex vivo* expansion relies on high-dose interleukin 2 (IL-2; 6000 IU ml^−1^)^3^. Numerous strategies to improve cellular fitness have demonstrated clinical efficacy, including *ex vivo* addition of various costimulatory molecules such as anti-4-1BB, which improves outgrowth of bulk effector CD8 TIL populations^6-8^. Additional strategies that have been explored include subset selection^1,9,10^ and genetic modification^11-13^; however, these approaches add technical complexity, time, and cost.

We hypothesized that a simplified cytokine reprogramming strategy applied early in *ex vivo* culture could generate functionally superior TILs that may improve overall response to this therapy. Prior studies in our lab revealed that helper Type-17 (Th17)-polarized CD4^+^ T cells mediate durable disease control in preclinical models and engaged host B cells for long-term protection^14,15^. Building on this work, we extended our translational investigations to human TIL, whereby we applied a single pulse of Type 17-associated cytokines at culture initiation, all currently available in GMP-grade formats, followed by low-dose IL-2 (200 IU mL^−1^) maintenance. We term this reprogrammed product polarized TIL, or “PolarTIL.”

Here, we demonstrate that PolarTIL represents a distinct cellular state with enhanced therapeutic efficacy. In a model of anti-PD-1 resistant murine melanoma, PolarTIL eradicated established disease and conferred durable, TIL-dependent systemic immunity, whereas conventionally expanded cultures were ineffective. In the human setting, PolarTIL derived from patients with melanoma and other resectable cutaneous malignancies achieved five-fold greater expansion than patient-matched TIL generated under standard, high-dose IL-2 conditions, exhibited superior cytotoxicity against autologous tumors, and increased intrinsic effector cytokine production. These products contained proportionally more CD103^+^ effector populations, fewer CD4 + regulatory T cells, and expressed features of tissue-resident memory differentiation^16,17^. Unexpectedly, TGF-β1 proved indispensable for efficacy, uncovering an unanticipated role for this cytokine in programming potent antitumor immunity. Together, these findings establish PolarTIL as a novel cell therapy product with superior expansion, favorable phenotypic properties and augmented function in comparison to conventional TIL.

## Results

### Polarization of murine melanoma TIL induces durable antitumor immunity in vivo.

To more closely model human TIL cultures, we generated TIL directly from established tumors, rather than using splenocytes. Subcutaneous B16F10 melanoma tumors were implanted in Pmel-1 mice (Thy1.1^+^), which express a gp100-specific transgenic T cell receptor on CD8 T cells^18^ (Fig. 1a). Once melanoma tumors were established, they were harvested and infiltrating CD45^+^ immune cells were isolated. The CD45^+^ cells were expanded *ex vivo* using either a conventional high-dose (HD) IL-2 protocol or the PolarTIL cytokine reprogramming strategy (see Methods) for 10-14 days. Subsequently, TIL were adoptively transferred into congenic, C57BL/6J mice (Thy1.2^+^) bearing B16KVP tumors, which express a high-affinity gp100 epitope recognized by Pmel-1 CD8^+^ T cells^19^.

PolarTIL cultures expanded to significantly greater numbers than HD IL-2 expanded counterparts (Fig. 1b). Upon adoptive transfer, PolarTIL mediated robust and durable control of tumor burden for more than 100 days, whereas HD IL-2-expanded TIL resulted in a modest trend toward tumor growth delay that did not reach statistical significance (Fig. 1c-d). Surviving PolarTIL-treated mice were then subjected to further tumor challenge to assess the durability of the response (Fig. 1e). Notably, mice achieving complete response following PolarTIL treatment were fully protected against contralateral subcutaneous rechallenge with B16KVP melanoma, indicating the establishment of durable systemic antitumor immunity compared to their naïve counterparts (Fig. 1f).

Given the recent work on host engagement in Th17 ACT^14^, we next assessed the contribution of persisting donor PolarTIL to protection following tumor rechallenge. Donor cells were selectively deleted in a subset of survivors from the same cohort of mice using an anti-Thy1.1 antibody, while control animals received isotype antibody. Efficient depletion was achieved, confirmed by near-complete absence of Thy1.1^+^ donor PolarTIL cells across multiple tissues, including blood, tumor-draining lymph nodes (TDLN), spleens, skin at the original tumor site, and lungs (Fig. S1). On day 6 following depletion, cured mice were challenged intravenously with parental B16F10 cells to induce pulmonary metastases. Animals retaining donor PolarTIL exhibited a marked reduction in lung metastatic burden, whereas depletion of donor cells abrogated this protection, resulting in metastatic outgrowth comparable to control animals (Fig. 1g-h). Thy1.1^+^ donor PolarTIL cells primarily occupied the TDLN of cured mice but were also present at lower percentages in the blood, lungs, skin at the original tumor site, and spleens (Fig. 1i).

Together, these findings demonstrate that PolarTIL mediate potent and durable antitumor immunity and that continued persistence of the transferred cells is required for protection against tumor recurrence and metastatic dissemination. Given this robust and durable *in vivo* activity, we next examined whether PolarTIL expansion could similarly enhance the yield and functional quality of human TIL derived from patient tumors.

#### PolarTIL improves human TIL yield and quality

Building on the durable antitumor activity observed in murine melanoma, we next applied the PolarTIL strategy to early *ex vivo* expansion of human TIL derived from surgically resected tumors from patients with cutaneous malignancies. Patient samples included melanoma (n = 26), Merkel cell carcinoma (n = 4), basal cell carcinoma (n = 1), and squamous cell carcinoma (n = 1, patient characteristics summarized in Table 1). Tumors were mechanically dissociated into small fragments and cultured under either conventional HD IL-2 conditions or the PolarTIL strategy (Fig. 2a; see Methods).

Consistent with murine findings, PolarTIL cultures generated significantly greater numbers of TIL within two weeks of culture (Fig. 2b) and on average yielded 5-fold higher cumulative cell numbers over time (Fig. 2c) compared with HD IL-2 cultures. We successfully expanded TIL from 87.5% of samples with the PolarTIL strategy, while the HD IL-2 strategy was only successful 73.3% of the time. The relative proportion of CD8^+^ and CD4^+^ T cells was comparable between expansion strategies (Fig. 2d). PolarTIL did not exhibit increased expression of inhibitory or costimulatory receptors, including PD-1, Lag-3, Tim-3, ICOS, and CD28 (Fig. 2e-i). However, PolarTIL cultures contained significantly fewer CD4^+^CD25^+^ FoxP3^+^ T regulatory cells (Fig. 2j,k), indicating improved cellular composition without skewing overall lineage balance. To assess killing ability, expanded TIL were challenged against autologous tumor cells using a real-time impedance-based cytotoxicity assay. PolarTIL mediated more rapid tumor cell lysis than HD IL-2 TIL (Fig. 2l-m).

Functionally, PolarTIL produced significantly more pro-inflammatory cytokines and cytotoxic molecules than HD IL-2 TIL as measured in the supernatant of reactivated cells (Fig. 3a). Specifically, IL-2, interferon-g (IFNg), granzyme B, perforin, tumor necrosis factor (TNF)-related apoptosis-inducing ligand (TRAIL), and IL-9 are produced at significantly higher levels by PolarTIL compared to HD IL-2 TIL (Fig. 3b-h). HD IL-2 TIL produce slightly more IL-6 than PolarTIL (Fig. 3i). Additionally, PolarTIL produce more chemokines associated with effector cell trafficking, including CCL28 (Fig. 3j, binding partners CCR3 and CCR10), CXCL11 (I-TAC, Fig. 3k, binding partner CXCR3), CCL5 (RANTES, Fig. 3l, binding partners CCR1, CCR3, CCR5). PolarTIL also showed a trend towards higher levels of sCD40L (Fig. 3m), the binding partner of CD40 on APCs such as B cells, and s4-1BB, consistent with antigen-induced T cell activation^20,21^ (Fig. 3n).

Together, these findings demonstrate that PolarTIL expansion enhances both the yield and functional quality of human TIL while limiting regulatory T cell outgrowth.

### PolarTIL expansion generates a transcriptionally and clonally distinct TIL state.

To define the molecular features underlying this distinct functional state, we next performed integrated single-cell transcriptional and T cell receptor (TCR) profiling of TIL from five patients expanded under HD IL-2 or PolarTIL conditions, along with matched pre-expansion TIL from three of these patients (Fig. 4a). Harmony integration identified 18 transcriptionally distinct clusters across all samples (Fig. 4b). Comparative analysis revealed that HD IL-2-expanded TIL clustered closely with pre-expansion TIL, whereas PolarTIL represented a distinct transcriptional population, consistent with induction of a reprogrammed cellular state (Fig. 4c). Pre-expansion Day 0 TIL harbored a high proportion of exhausted CD8^+^ T cells, while HD IL-2 TIL contained CD8^+^ T cells at various levels of exhaustion (Fig. 4d). The PolarTIL strategy unsurprisingly expanded an enhanced percentage of CD4^+^ Th17 cells, along with *GZMB*^+^ CD8^+^ T cells with a tissue resident memory (TRM) transcription profile (*ITGAE^+^ ITGA1^+^ CXCR6^+^*). There was also an increased proportion of CD8^+^ TRM cells expressing *ZNF683* (Hobit), which have been identified as cancer-specific resident memory T cells in other malignancies^22^.

To assess how expansion strategy influenced clonal architecture, we examined TCR diversity using clonal rarefaction analysis. PolarTIL exhibited reduced clonal diversity compared with HD IL-2-expanded TIL (Fig. 4e), indicating preferential expansion of selected clones. TCR clonal overlap was highest between HD IL-2 TIL and pre-expansion Day 0 TIL (Morisita Index of 0.511), while PolarTIL had less overlap with pre-expansion TIL (Morisita Index of 0.114). However, partial clonal overlap between HD IL-2 and PolarTIL products (Morisita Index of 0.416) indicated retention of shared antigen specificities despite divergent expansion trajectories (Fig. 4f). Comparison against previously described tumor- and virus-specific gene signatures^23,24^ revealed that PolarTIL clones were enriched for tumor-reactive gene signatures while HD IL-2 TIL more highly expressed a virus-reactive signature (Fig. 4g).

Differential gene expression analysis identified 408 genes differentially expressed between HD IL-2 and PolarTIL (Fig. 4h). PolarTIL were enriched for transcripts associated with chemotactic migration and cytokine signaling, including *CCR4, CCR7*, *IL7*, *IL6R* as well as *GPR183*, which encodes for an oxysterol receptor that has been implicated in improved T cell infiltration into solid tumors^25,26^. T-B cell crosstalk and antigen presentation associated genes (*CD86*, *CD40L*, *CXCL13, CIITA, HLA-DRA*) were also enriched in PolarTIL. In contrast, HD IL-2 preferentially expressed genes associated with TGF-β signaling (*SMAD3*, *TGFBR3*), stem-associated programs (*TCF7*), and exhaustion-associated pathways (*ID2*, *SLAMF7*, *IRF4*). Genes associated with Th1 and Th2 differentiation (*CXCR3, HAVCR1*) were also increased under HD IL-2 conditions, whereas PolarTIL exhibited elevated expression of *SOX5*, a transcription factor linked to STAT3 signaling and Type 17 programs. Notably, PolarTIL were enriched for resident-memory-associated genes, including *ITGAE* (CD103) and *RUNX2*^17^.

Gene set enrichment analysis further supported a metabolic and biosynthetic shift in PolarTIL, with enrichment of pathways related to RNA translation and oxidative phosphorylation, whereas HD IL-2-expanded TIL enriched pathways associated with cellular homeostasis and glycolytic metabolism (Fig. 4i). In summary, these data demonstrate that PolarTIL expansion drives a transcriptionally and clonally distinct TIL state, characterized by selective clonal amplification and enrichment of programs associated with tissue localization and durable immune function.

#### TGF-β1 is a central driver of the PolarTIL functional state

The enrichment of resident memory–associated transcriptional programs in PolarTIL prompted us to validate these findings at the protein level. We therefore profiled matched pre-expansion TIL and post-expansion products generated under HD IL-2 or PolarTIL conditions from five melanoma patients for expression of markers associated with tissue residency and neoantigen-reactive CD8^+^ T cells, including CD103, CXCR6, and CD39^27-31^. CD103^+^CD39^+^ and CD103^+^CXCR6^+^ CD8^+^ T cells were detectable within pre-expansion TIL, consistent with the presence of resident and neoantigen-like reactive populations in situ, but these double-positive populations did not significantly increase following HD IL-2 expansion. In contrast, both populations were markedly enriched following PolarTIL expansion, indicating preferential amplification of resident memory-like CD8^+^ T cells (Fig. 5a-d).

To determine which components of the polarization strategy were responsible for this phenotypic reprogramming, we next generated PolarTIL cultures in which single cytokine components were systematically removed. Removal of IL-1β, IL-6, IL-21, αIFNg or αIL-4 from PolarTIL modestly reduced overall TIL expansion, whereas removal of TGF-β1 did not significantly alter final cell yield (Fig. S2a). Across all cytokine subtraction conditions, CD4 + TIL composition shifted toward increased CXCR3^+^CCR6^−^ Th1-like cells and reduced CXCR3^−^CCR6^+^ Th17-like cells, with removal of TGF-β1 driving the most pronounced reduction in Th17-associated populations (Fig. S2b,c). Removal of all cytokines except TGF-β1 increased the frequency of FoxP3^+^CD25^+^CD4^+^ regulatory T cells (Tregs) and CXCR5^+^ PD-1^+^ T follicular helper (Tfh)-like cells (Fig. S2d,e).

Given prior evidence implicating TGF-β1 signaling in tissue-resident memory differentiation^32,33^, we next examined resident memory-associated phenotypes in CD8^+^PolarTIL expanded with or without TGF-β1. Strikingly, exclusion of TGF-β1 from the PolarTIL strategy reduced the frequency of CD8^+^ TIL co-expressing CD103 and CD49a to below those observed with HD IL-2 TIL expansion, whereas removal of other PolarTIL components did not elicit comparable effects (Fig. 5e,f). Consistent with transcriptomic analysis, protein expression of RUNX2 was increased in PolarTIL relative to HD IL-2 TIL cultures, but this increase was abolished when TGF-β1 was excluded (Fig. 5g,h).

To assess whether TGF-β1 alone was sufficient to recapitulate the PolarTIL phenotype, we expanded TIL using TGF-β1 and IL-2 in isolation. Although overall expansion was preserved, this condition resulted in a marked increase in Tregs, indicating that TGF-β1 is necessary but not sufficient to generate the PolarTIL state (Fig. S2g,h). These findings suggested that TGF-β-dependent programming may be required not only for PolarTIL phenotype, but also for antitumor function in vivo.

### Loss of TGF-β1 during polarization abrogates the antitumor activity of PolarTIL in vivo.

We next tested whether TGF-β1-dependent programming was required for the superior antitumor activity of PolarTIL. Murine TIL were generated from B16F10 tumors established in Pmel-1 mice and expanded under HD IL-2 conditions, PolarTIL conditions, or PolarTIL conditions lacking TGF-β1 (Fig. 6a). Consistent with human findings, exclusion of TGF-β1 did not impair expansion of PolarTIL (Fig. 6b).

Phenotypic analysis revealed that murine PolarTIL expressed elevated CD103 levels relative to HD IL-2-expanded TIL, and this enrichment was lost when TGF-β1 was excluded from the polarization strategy (Fig. 6c,d). Following adoptive transfer into mice bearing B16KVP tumors, only PolarTIL generated under full polarization conditions engrafted detectably in the peripheral blood by day 11 post-ACT, whereas HD IL-2 TIL and TGF-β1-deficient PolarTIL failed to persist (Fig. 6e). Functionally, only fully polarized PolarTIL could regress and prevent the growth of melanoma, and this antitumor activity was completely ablated when TGF-β1 was omitted from the polarization strategy (Fig. 6f-g).

Together, these findings identify TGF-β1 as a critical determinant of the PolarTIL transcriptional program, resident memory–associated phenotype, *in vivo* persistence, and antitumor efficacy, revealing a context-dependent role for this pathway in programming durable antitumor immunity.

## Discussion

Adoptive transfer of tumor-infiltrating lymphocytes (TIL) can induce durable clinical responses in patients with solid malignancies, yet therapeutic benefit does not extend to every patient and is potentially diminished in individuals who progress on immune checkpoint blockade (ICB) prior to cell therapy^1-5^, suggesting that the TIL product that these patients receive may not have an optimal phenotype to drive response. These clinical observations highlight intrinsic limitations in prevailing TIL manufacturing paradigms, which prioritize numerical expansion under sustained high-dose interleukin-2 (IL-2) with limited consideration of long-term functional programming^6-8^. Here, we demonstrate that transient cytokine reprogramming early during ex vivo culture is sufficient to redirect TIL fate, generating a distinct product, PolarTIL, with enhanced persistence and antitumor efficacy.

A brief pulse of Type 17-associated cytokines at culture initiation yielded PolarTIL with multiple advantageous properties. Compared with conventional high-dose IL-2 expansion, PolarTIL achieved greater overall yield, limited outgrowth of regulatory T cells, increased effector cytokine production, and superior cytotoxicity against autologous tumor targets. Notably, these improvements were achieved without genetic manipulation, prolonged cytokine exposure or selection of predefined T cell subsets, distinguishing this approach from alternative strategies that increase technical complexity, cost and translational encumbrance^1,9-13^. These findings indicate that early instructional signals, rather than sustained expansion conditions alone, are critical determinants of TIL functional fitness.

Single-cell transcriptomic and paired T cell receptor (TCR) profiling revealed that PolarTIL occupy a transcriptional and clonal state distinct from both pre-expansion and conventionally expanded TIL. PolarTIL preferentially amplified selected TCR clones and were enriched for transcriptional programs associated with tissue localization, immune cell interaction, and biosynthetic activity. The PolarTIL expansion strategy also enriched cellular compartments with CD4^+^ Th17 and CD8^+^ granzyme B (*GZMB*)^+^ tissue-resident memory transcriptional profiles. Clonally, PolarTIL exhibited less diversity and less overlap with pre-expansion TIL than HD IL-2 TIL. Despite being less clonal, PolarTIL clones contain more cells with a tumor-specific gene signature while HD IL-2 clones have more virus-specific, or bystander, gene signature. These data indicate that the PolarTIL strategy may be preferentially expand cells bearing a tumor-reactive signatures, though further investigation is needed to determine why this may be occurring.

In contrast, TIL expanded under high-dose IL-2 conditions exhibited increased expression of Tex gene profiles as well as glycolytic programs. A striking and unexpected difference emerged in the TGF-β1 axis itself. HD IL-2 TIL upregulated genes associated with TGF-β signaling, including *SMAD3* and *TGFBR3*, despite never being exposed to exogenous TGF-β1, whereas PolarTIL, which received a defined TGF-β1 pulse at culture initiation, displayed lower TGF-β-pathway transcripts. We interpret this as a pulse-induced desensitization: a single early dose of TGF-β may render PolarTIL refractory to the sustained TGF-β signaling that dominates the tumor microenvironment, a resilience that could contribute to their durable activity *in vivo*. This mechanism remains to be formally tested.

Mechanistically, PolarTIL were enriched for *SOX5*, a transcription factor linking STAT3 signaling with RORgT expression and Type 17 differentiation programs^34^, together with transcriptional networks associated with tissue localization and functional persistence^17,33,35^. RUNX2 and RUNX3 can regulate expression of CD103 and CD49a, defining tissue-resident CD8^+^ T cells poised for cytotoxic activity in non-lymphoid tissues^16,17^. Consistent with this framework, PolarTIL exhibited increased frequencies of CD103^+^CD39^+^ and CD103^+^CXCR6^+^ CD8^+^ T cells, populations associated with neoantigen specificity and tumor reactivity across multiple solid malignancies^9,27-29,31,36^. While tissue-resident memory (TRM) features emerged as a prominent component of the PolarTIL phenotype, our data support a broader model in which early cytokine programming promotes a durable effector state that may encompass both resident and circulating memory populations.

Unexpectedly, systematic dissection of the polarization cocktail identified TGF-β1 as a central driver of the PolarTIL phenotype and functional state. Although TGF-β signaling is classically associated with immunosuppression and resistance to immunotherapy within established tumors^37,38^, we found that transient exposure during early TIL programming was required for acquisition of resident memory-associated features and durable antitumor activity.

Removal of TGF-β1 abolished induction of RUNX2, reduced enrichment of CD103^+^ effector populations, impaired persistence following adoptive transfer, and eliminated therapeutic efficacy in vivo. These findings underscore the context-dependent effects of TGF-β signaling and demonstrate that temporally restricted exposure during *ex vivo* programming in combination with other cytokines that drive inflammatory pathways can impart durable functional advantages rather than suppression^32,33^.

PolarTIL displayed transcriptional enrichment of genes in the oxidative phosphorylation and RNA translation pathways. Reliance on oxidative metabolism has been linked to enhanced persistence, apoptotic resistance, and antitumor efficacy in Th17-like cells^39-41^, providing a plausible metabolic basis for the sustained activity observed *in vivo*. In parallel, PolarTIL expressed elevated *CD40L* and *CXCL13* transcripts (and protein, in the case of CD40L), as well as multiple chemokines, suggesting an increased capacity for immune crosstalk following transfer. Prior studies have demonstrated that polarized Th17 cells engage host B cells to support durable antitumor immunity including induction of tumor-specific antibody responses^14,15^, raising the possibility that human PolarTIL may similarly coordinate multi-lineage host immune responses after infusion in patients treated with this product, which will be investigated in future work. Additionally, the observed increase in IL-2 secretion may indicate that PolarTIL would require less post-administration IL-2 support, a further way that this product may reduce the cost of this therapy.

In summary, this work establishes PolarTIL as a distinct therapeutic product generated through transient cytokine reprogramming early during ex vivo culture. To note, the cytokines used in this study are already available in GMP grade formats, making this strategy easily translatable. By uncovering a context-dependent role for TGF–β1 in programming durable antitumor immunity, these findings challenge prevailing assumptions regarding TGF-β signaling in cancer and provide a practical framework for improving TIL efficacy in the setting of checkpoint resistance. Clinical translation of PolarTIL is actively underway to provide a novel cellular therapy alternative with potential advantages and potency in patients with refractory solid tumors.

## Methods

### Cell culture conditions

Human and mouse TIL were cultured *in vitro* in complete media (CM) composed of RPMI 1640 (Corning) supplemented with 10% heat inactivated (HI) FBS (Atlas Biologics), 1% pen/strep (Corning), HEPES (Gibco), non-essential amino acids (Gibco), sodium pyruvate (Gibco), and 2-mercaptoethanol (Gibco). Cytokines for TIL manufacturing included recombinant human (rh) IL-2 (NCI BRB Preclinical Biologics Repository), 10 ng/mL rhIL-1β, 100 ng/mL rhIL-6 (NCI BRB Preclinical Biologics Repository), 100 ng/mL rhIL-21, 30 ng/mL rhTGF-β1, 20 ng/mL rhIL-23 or 20 ng/mL recombinant mouse (rm) IL-23, 5 μg/mL anti-human or 10 μg/mL anti-mouse IFNγ (clones B27 or XMG1.2, respectively), 5 μg/mL anti-human or 10 μg/mL anti-mouse IL-4 (clones MP4-25D2 or 11B11, respectively), and 30 ng/mL anti-human or anti-mouse CD3 (clones OKT3 or 145-2C11, respectively).

### Mice and Cell Lines

Female C57BL/6J (Thy1.2+) and pmel-1 (Thy1.1+) mice aged 6 to 8 weeks were obtained from Jackson Laboratories and housed and maintained in specific pathogen-free animal facilities at Emory University. All experiments were performed under Emory University IACUC-approved protocol #PROTO201900225. Experimental hosts were female, and donor animals for adoptive transfer were male or female. All experiments were carefully monitored in accordance with IACUC guidelines to minimize suffering. Euthanasia was performed using CO asphyxiation followed by cervical dislocation. Housing conditions were a dark/light cycle of 12h/12h and a humidity of approximately 55%.

The B16F10 (ATCC) murine melanoma cell line was maintained in CM. The B16KVP murine melanoma cell line (gift from Dr. Nicholas Restifo) was cultured in CM containing 10 μg/mL blasticidin S. Human tumor cultures expanded from single-cell suspensions of tumor digests were maintained in CM containing reduced HI-FBS (2%). All cell cultures were maintained at 37°C with 5% CO2 in incubators.

### Murine TIL Generation

Female pmel-1 mice aged 6 to 8 weeks were injected subcutaneously with the murine melanoma cell line B16F10, and tumor growth was monitored for 7 to 10 days until tumors reached an average diameter of 10 mm. Mice were then euthanized, and tumors were harvested and pooled. Tumors were digested using the gentleMACS Mouse Tumor Dissociation Kit (Miltenyi) according to the manufacturer’s protocol. Single-cell suspensions were passed through a 40 μm filter and counted using the EVE Plus cell counter (NanoEntek). Positive selection of CD45 + TILs was performed using mouse CD45 (TIL) MicroBeads (Miltenyi) according to the manufacturer’s protocol. Positively selected cells were split between high-dose IL-2 medium or PolarTIL medium and seeded at a density of 1 × 10^6 cells/mL in multiwell plates. On day 5 post-activation, half of the medium was replaced with fresh medium containing either 6,000 IU/mL rhIL-2 (HD IL-2 condition) or 200 IU/mL rhIL-2 and 10 ng/mL rmIL-23 (PolarTIL condition). Cells were split as needed to maintain a density of 1 × 10^6 cells/mL; all fresh medium contained the same cytokines as those added on day 5 post-activation. TIL cultures were expanded for 12 to 14 days before flow cytometric characterization and adoptive cell transfer.

### Adoptive Cell Therapy

C57BL/6J mice aged 6 to 8 weeks were injected subcutaneously with 5 × 10^5 B16KVP cells. Tumors were established for 6 days before lymphodepletion with 5 Gy total body irradiation. One day after irradiation, TIL cultures were harvested, washed twice, and resuspended at a dose of 1 to 10 × 10^6 cells per mouse. Cells were infused via tail vein, after which IL-2 complex (1.5 μg rhIL-2 + 7.5 μg anti-IL-2 [clone 5355, R&D Systems]) was administered via intraperitoneal injection. Mice received additional IL-2 complex on days 2 and 4 post-ACT. Tumor measurements (length × width) were recorded twice weekly, and mice were euthanized if tumors exceeded 20 mm in diameter.

Surviving mice with no remaining tumor were rechallenged subcutaneously on the opposite flank with 5 × 10^5 B16KVP cells on day 59 post-ACT, and tumor growth was monitored. Remaining survivors were then injected intravenously with 500 μg anti-Thy1.1 antibody (clone 19E12, BioXCell) or mouse IgG2a isotype control (clone C1.18.4, BioXCell) to deplete remaining Thy1.1 + pmel-1 cells on day 122 post-ACT. Mice were then injected intraperitoneally with 250 μg anti-Thy1.1 or isotype control on day 125 post-ACT and were inoculated intravenously with 2 × 10^5 B16F10 cells the following day. Additional intraperitoneal injections of 250 μg anti-Thy1.1 or isotype control were performed weekly until mice were euthanized for biodistribution analysis on day 147 post-ACT. Blood, lungs, spleen, skin at the original tumor site, and tumor-draining lymph nodes were collected from remaining mice, processed into single-cell suspensions, and stained for flow cytometry.

### Human TIL Generation

Human tumor specimens were surgically resected from patients, deidentified after gross pathologic evaluation, and distributed for research by the Cancer Tissue and Pathology shared resource of Winship Cancer Institute of Emory University. The research protocol was reviewed and approved by Emory University’s Institutional Review Board (IRB), protocol #2025P010983. All donors gave written informed consent for the collection and use of samples. This study design and conduct complied with the regulations on the use of human study participants and was conducted in accordance with the Declaration of Helsinki. Donor patient demographics are listed in Table 1.

Tumor samples were cut precisely into pieces approximately 3 mm in diameter with a scalpel blade. Pieces were placed individually into wells of a 24-well tissue culture plate or 24-well G-Rex plate (Wilson Wolf), or three pieces were placed into a G-Rex 6M plate. Medium with HD IL-2 conditions, PolarTIL conditions, or PolarTIL conditions without specific cytokines (for subtraction experiments) was added to each well (2 mL for 24-well plates, 10 mL for G-Rex 6M plates). On day 5 post-activation, the respective medium was either replaced (tissue culture plate) or added (G-Rex). HD IL-2 cultures received 6,000 IU/mL rhIL-2, and PolarTIL cultures received 200 IU/mL rhIL-2 and 10 ng/mL rhIL-23. TILs were expanded for 2 to 3 weeks total. Cultures in tissue culture plates were split as needed when confluence was reached, and replenishment medium contained the same respective cytokines as those added on day 5. G-Rex cultures were left undisturbed until harvest. At harvest, cells were collected and counted. Some cells were used for flow cytometry or cytolysis assays, and the remaining cells were cryopreserved for future assays.

Remaining tumor was digested with the Human Tumor Dissociation Kit (Miltenyi) according to the manufacturer’s instructions. Single-cell suspensions were passed through a 70 μm filter and counted. Cells were then either processed for flow cytometry, cryopreserved for future use, or plated in a tissue culture flask containing RPMI with reduced serum (2%) for tumor culture generation. Tumor cultures were passaged as needed and then used for cell cytolysis assays with patient-matched TIL samples.

### Flow cytometry

For all flow cytometry experiments, cells were washed in PBS and resuspended in live/dead stain (Zombie Aqua or Zombie UV, BioLegend) for 10 minutes at room temperature (RT). After washing with FACS buffer (PBS + 2% HI-FBS), cells were resuspended in an extracellular staining master mix consisting of antibodies diluted in FACS buffer (antibody information listed in Table S1) for 10 minutes at RT in the dark. After incubation, cells were washed twice with FACS buffer and resuspended in FoxP3 Fixation/Permeabilization Buffer (eBioscience) for 30 minutes at RT in the dark. Cells were then washed once with 1× permeabilization buffer (eBioscience) and resuspended in a master mix containing intracellular antibodies diluted in permeabilization buffer for 30 minutes at RT in the dark. Cells were then washed twice, resuspended in FACS buffer, and run on a 5-laser Cytek Aurora spectral flow cytometer.

### Cell cytolysis assay

Tumor cell cultures generated from patient samples were incubated with 0.05% trypsin-EDTA (Corning) until cells detached from the flask. Cells were centrifuged, washed, resuspended in CM, and enumerated. Cell cytolysis assays were performed using the xCELLigence MP instrument and Real-Time Cell Analysis (RTCA) Software (Agilent). E-Plate 96-well plates were first blanked with CM alone, and then 1 × 10^4 tumor cells were added to each well. The E-Plate was placed in the instrument, and continuous impedance measurements were recorded every 15 minutes for approximately 24 hours or until logarithmic cell growth was observed. At that point, patient-matched TILs expanded under either HD IL-2 or PolarTIL conditions were added to wells at effector-to-target (E:T) ratios of 20:1, 10:1, and 5:1. Impedance measurements were then recorded until the impedance of control (tumor-only) wells began to decrease, indicating the growth limit of tumor cells in the wells. The experiment was then concluded, and RTCA software was used to calculate the percentage of specific lysis of target tumor cells.

### Multiplex biomarker assay

Expanded HD IL-2 TILs and paired PolarTILs from patients (n = 5) were thawed from cryopreservation and rested overnight in medium. TILs were then counted and plated in a 96-well U-bottom plate (2 × 10^5 cells per well) containing medium with 30 ng/mL anti-human CD3 (OKT3) and either 6,000 IU/mL rhIL-2 (HD IL-2) or 200 IU/mL rhIL-2 (PolarTIL). Three days after plating, culture supernatant was collected, clarified by centrifugation, and frozen at −20°C. Supernatant was analyzed with a 96-plex Discovery Assay Array (HD96, Eve Technologies) on the Luminex 200 platform.

### Single-cell RNA sequencing

Samples of pre-expansion (day 0) tumor suspensions and patient-matched expanded TIL cultures were thawed from cryopreservation and rested overnight in CM at 37°C with 5% CO2. The following day, cells were counted and stained with Live/Dead stain (Zombie Aqua) in sterile PBS for 10 minutes at RT. After washing with sterile FACS buffer, cells were stained with anti-human CD3 (clone OKT3) and sorted for live CD3 + cells on a BD FACSAria II cell sorter. After sorting, isolated cells were fixed with a cell fixation kit (Parse Biosciences) and frozen according to the manufacturer’s protocol at −80°C until subsequent barcoding steps. Single-cell sequencing preparation was performed with the Evercode WT v2 or v3 Single Cell Whole Transcriptome and TCR kits (Parse Biosciences) according to the manufacturer’s protocol. This process generated pooled sublibraries of cells with unique individual barcodes from which cDNA was captured and amplified with Illumina TruSeq Read 2-specific primers. The resulting amplified cDNA was then used to generate whole-transcriptome and TCR sequencing libraries.

Whole-transcriptome libraries were generated by fragmentation of the cDNA, followed by end repair and A-tailing. An Illumina TruSeq Read 1 adapter was then ligated to the 5′ end of the DNA, after which the sequencing library was amplified with P5/P7 adapters and an additional barcode specific to each sublibrary. The resulting sequencing libraries were quantified and quality assessed using the Qubit 1X dsDNA High Sensitivity assay (Thermo Fisher), Bioanalyzer (Agilent), and KAPA qPCR. The pooled sublibraries were then sequenced with a NovaSeq S4 kit (Illumina) on a NovaSeq 6000 Sequencer (Illumina), generating approximately 100,000 reads per cell.

TCR libraries were generated by amplification of the subset of cDNA containing V(D)J segments in the CDR3 repertoire along with the addition of an Illumina adapter. Final amplification of this product, with the addition of P5 and P7 adapters and a fourth sublibrary-specific barcode, was performed, and the resulting sequencing libraries were quantified and quality assessed as described for the whole-transcriptome libraries above. Pooled sublibraries were sequenced with a NextSeq 2000 P2 XLEAP 300-cycle kit (Illumina), generating approximately 5,000 reads per cell.

### Data and Statistical Analyses

Flow cytometry analyses were performed with FlowJo v10 Software (BD). Graphing and statistical analyses were performed with GraphPad Prism 10 software (GraphPad Software, Inc.). Graphics were created using BioRender.

Whole-transcriptome single-cell raw data were processed through the Parse Pipeline v1.6 (Parse Biosciences) on Amazon Web Services (AWS), which identified cell barcodes and aligned sequencing reads to the human EMBL-EBI GRCh38 reference genome, generating a cell-by-gene count matrix. TCR single-cell raw data were subsequently processed through the pipeline, which built consensus contigs for each cell with annotations.

Processed data were normalized and filtered using the Seurat v5.3.8 R package in RStudio (Posit Software). Dimensionality reduction using Harmony batch correction was performed on data from two sequencing runs, followed by Louvain clustering. Cluster identities were analyzed and annotated by identifying the top 10 markers per cluster and then further refining annotations based on canonical T cell genes. Pipeline-processed TCR data were analyzed using the R package scRepertoire in RStudio. Clonal rarefaction analysis used the Shannon diversity index and a sample-size-based rarefaction and extrapolation curve. Clonal overlap between samples was calculated using the Morisita overlap index. Gene signature analyses were performed following previously published code using published datasets.

For additional analyses, pipeline-processed whole-transcriptome data were uploaded to BBrowserX Pro Software (Bioturing, Inc.). Dimensionality reduction using Harmony batch correction was performed on data from two sequencing runs, followed by Louvain clustering (resolution = 3; n_neighbors = 15). Differential gene expression (DEG) analysis on log-normalized data using the Wilcoxon method between pooled PolarTIL and HD IL-2 samples identified significantly different genes between groups. Gene set enrichment analysis using the DEG results evaluated Gene Ontology (GO) Biological Processes (2023); results were sorted by normalized enrichment score (NES).

## Supplementary Material

This is a list of supplementary files associated with this preprint. Click to download.

- Table1.docx
- Supp.Figures.docx

## Figures and Tables

**Figure 1:**
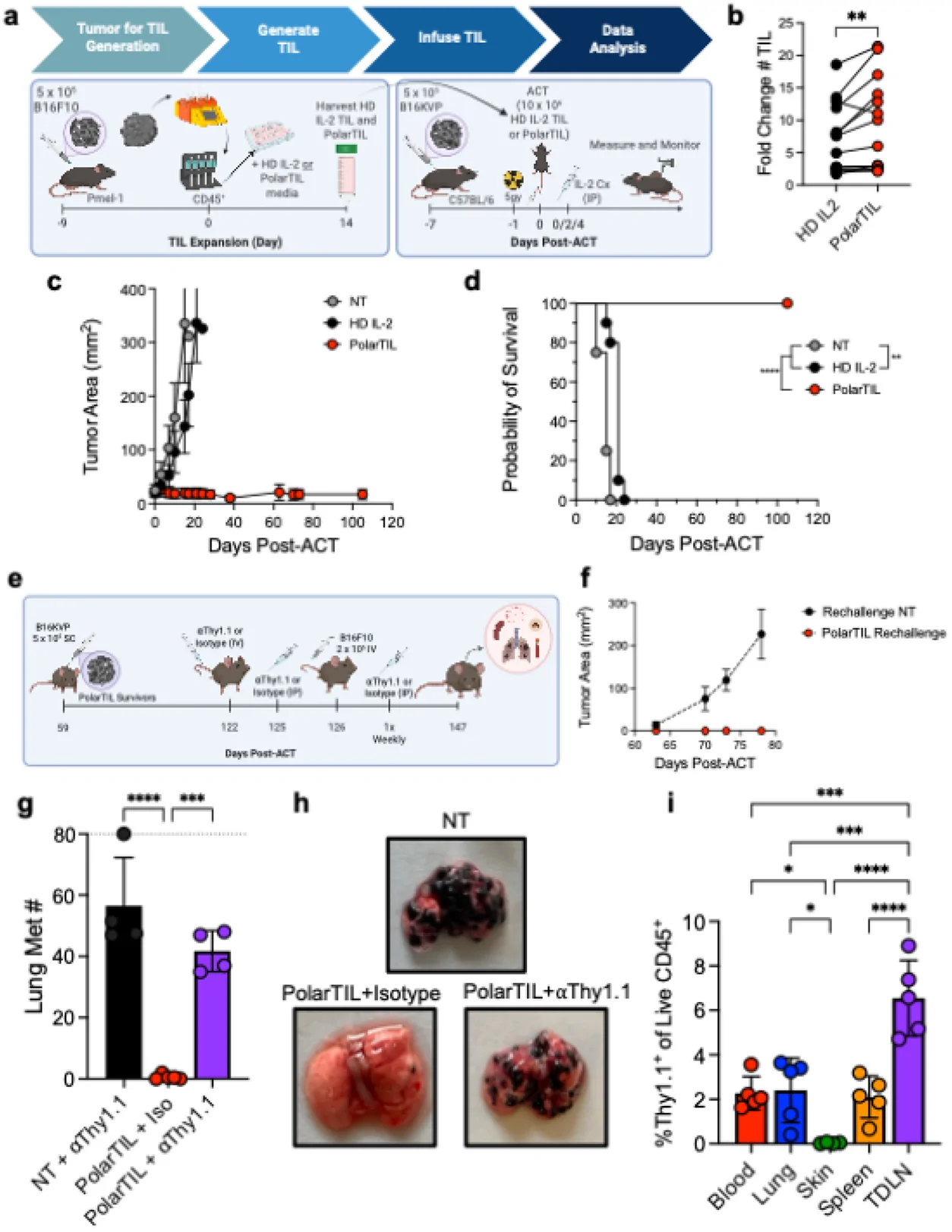
PolarTIL generation and treatment of murine melanoma. **a**, Initial TIL experiment schematic. CD45^+^ TIL were isolated from established B16F10 melanoma tumors harvested from Pmel-1 mice and cultured with media containing mCD3 and either HD IL-2 (6000 IU/mL IL-2) or PolarTIL cytokines (IL-1β, IL-6, IL-21, TGF-β1, 200IU/mL IL-2, mIL-4 and mIFNγ) for two weeks. Expanded TIL were adoptively transferred into lymphodepleted (5gy total body irradiation) C57BL/6J mice bearing established B16KVP tumors. TIL-therapy treated mice received IL-2 complex (rhIL-2 + L-2) on days 0, 2, and 4 post-adoptive cell therapy (ACT). Mice were monitored for tumor area until endpoint criteria was met. **b**, Murine PolarTIL expanded significantly more than murine HD IL-2 TIL (n=14, paired Wilcoxon test on raw TIL counts). **c**, Average tumor area (mm^2^) ± SD over time of no treatment (NT, gray, n=5), HD IL-2 TIL treated mice (black, n=10) and PolarTIL treated mice (red, n=10). **d**, Average probability of survival for each group (Kaplan-Meier with Holm-Šídák’s multiple comparisons test). **e**, Surviving mice rechallenge experimental schema. PolarTIL survivors from Fig. 1C were first rechallenged with a subcutaneous injection of B16KVP on the opposite flank from the original tumor site 59 days post-ACT. Survivors of this rechallenge were then depleted or not of Thy1.1+ donor TIL with IV followed by IP injections of either ^Thy1.1 depleting antibody or isotype. These mice were then IV injected with B16F10 tumor cells to induce lung metastases. Three weeks post-IV tumor challenge, the mice were sacrificed and organs (blood, spleen, lung, lymph nodes, and skin of the original tumor site) were collected. **f**, Average tumor area (mm^2^) ± SD of subcutaneous rechallenge B16KVP tumor. **g**, Lung metastases counts in no treatment control (black, n=4), PolarTIL survivors + Isotype antibody (red, n=5) and PolarTIL survivors + ^Thy1.1 depleting antibody (purple, n=4). Dashed line indicates limit of detection (80). **h**, Representative photos of lung metastases burden for each group. **i**, Detection of Thy1.1+ donor TIL in blood (red), lung (blue), skin of original tumor site (green), spleen (yellow), and tumor draining inguinal lymph nodes (purple) from PolarTIL survivor+Isotype mice (n=5, one-way ANOVA with Šídák’s multiple comparisons test). * = p<0.05, ** = p<0.01, *** = p<0.001, **** = p<0.0001.

**Figure 2:**
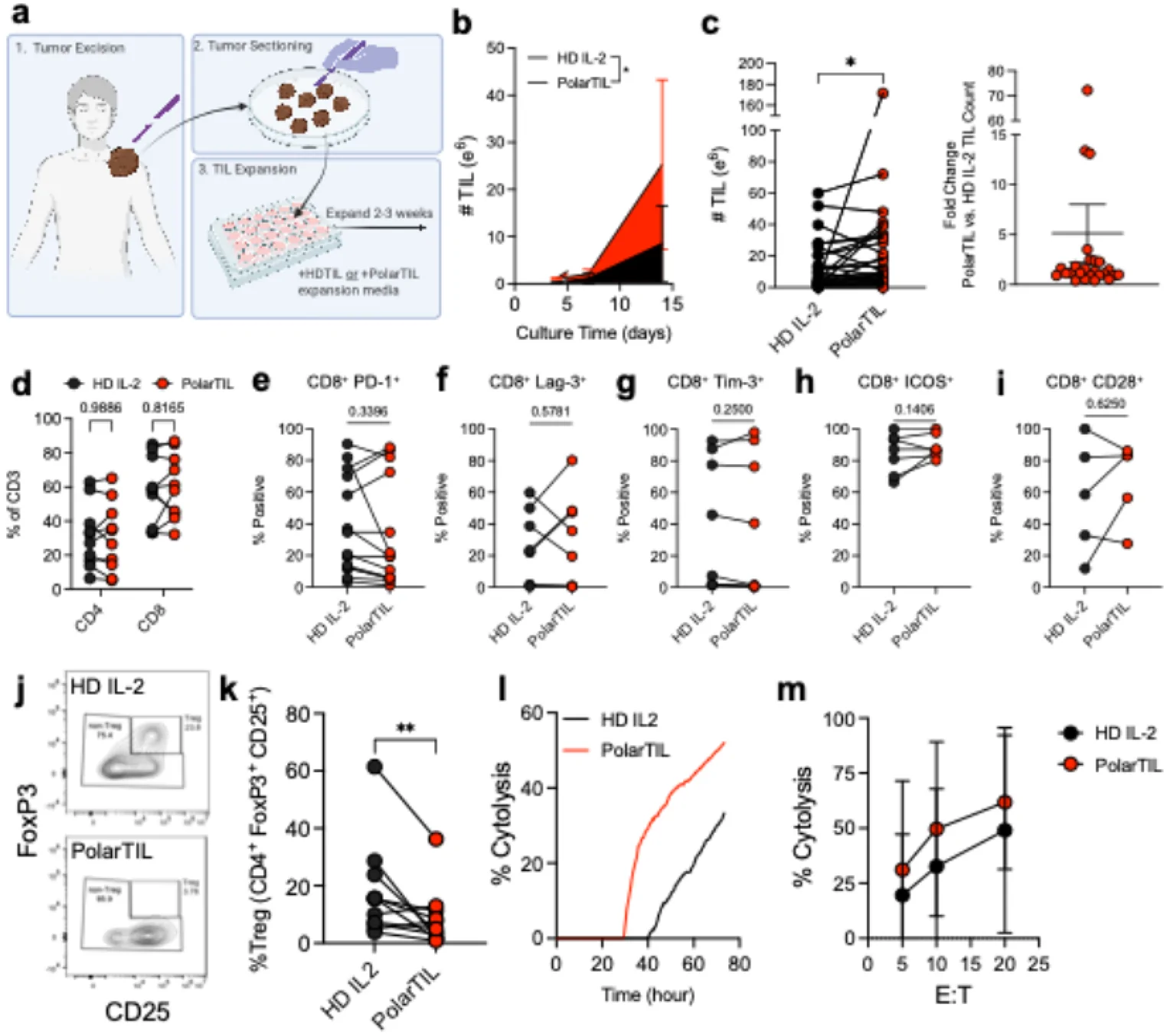
Patient PolarTIL have increased expansion capacity and cytotoxicity. **a**, Human patient TIL expansion workflow. Tumors samples are surgically excised and then sectioned into 2-3mm fragments. Individual fragments are placed into either a 24-well plate or Grex plate (1 piece/well), or larger Grex flask (2-5 pieces/flask) containing media with CD3 and either HD IL-2 (6000 IU/mL IL-2) or PolarTIL cytokines (IL-1β, IL-6, IL-21, TGF-β1, 200 IU/mL IL-2, hIL-4 and hIFNγ) for 2-3 weeks. **a**, PolarTIL (red) versus HD IL-2 TIL (black) expansion over time; n=9. **c**, Final TIL count at the end of culture compared between PolarTIL and HD IL-2 TIL (n=47; Wilcoxon matched-pairs signed rank test). **d**, Distribution of CD4^+^ and CD8^+^ of total CD3^+^ T cells in HD IL-2 TIL and PolarTIL cultures as analyzed by flow cytometry (n=10, 2-way ANOVA with Šídák’s multiple comparisons test). Percent PD-1^+^ (**e**), Lag-3^+^ (**f**), Tim-3^+^ (**g**), ICOS^+^ (**h**), and CD28^+^ (**i**) of CD8^+^ TIL per group as assessed by flow cytometry (n=14, Wilcoxon matched-pairs signed rank test). **j**, Representative flow cytometry plots of CD25 (x-axis) vs. FoxP3 (y-axis) on CD4+ HD IL-2 TIL (top) or PolarTIL (bottom). Double positive populations are denoted as Tregs. **k**, Percent FoxP3+CD25+CD4+ Tregs of HD IL-2 TIL and PolarTIL (n=12, Wilcoxon matched-pairs signed rank test). **l**, Representative % cytolysis curve over time for HD IL-2 TIL (black) and PolarTIL (red) against autologous tumor in Xcelligence assay. **m**, average ± SD percent cytolysis at different effector to target (E:T) ratios for HD IL-2 TIL and PolarTIL against autologous tumor in Xcelligence assay (n=2). * = p<0.05, ** = p<0.01.

**Figure 3:**
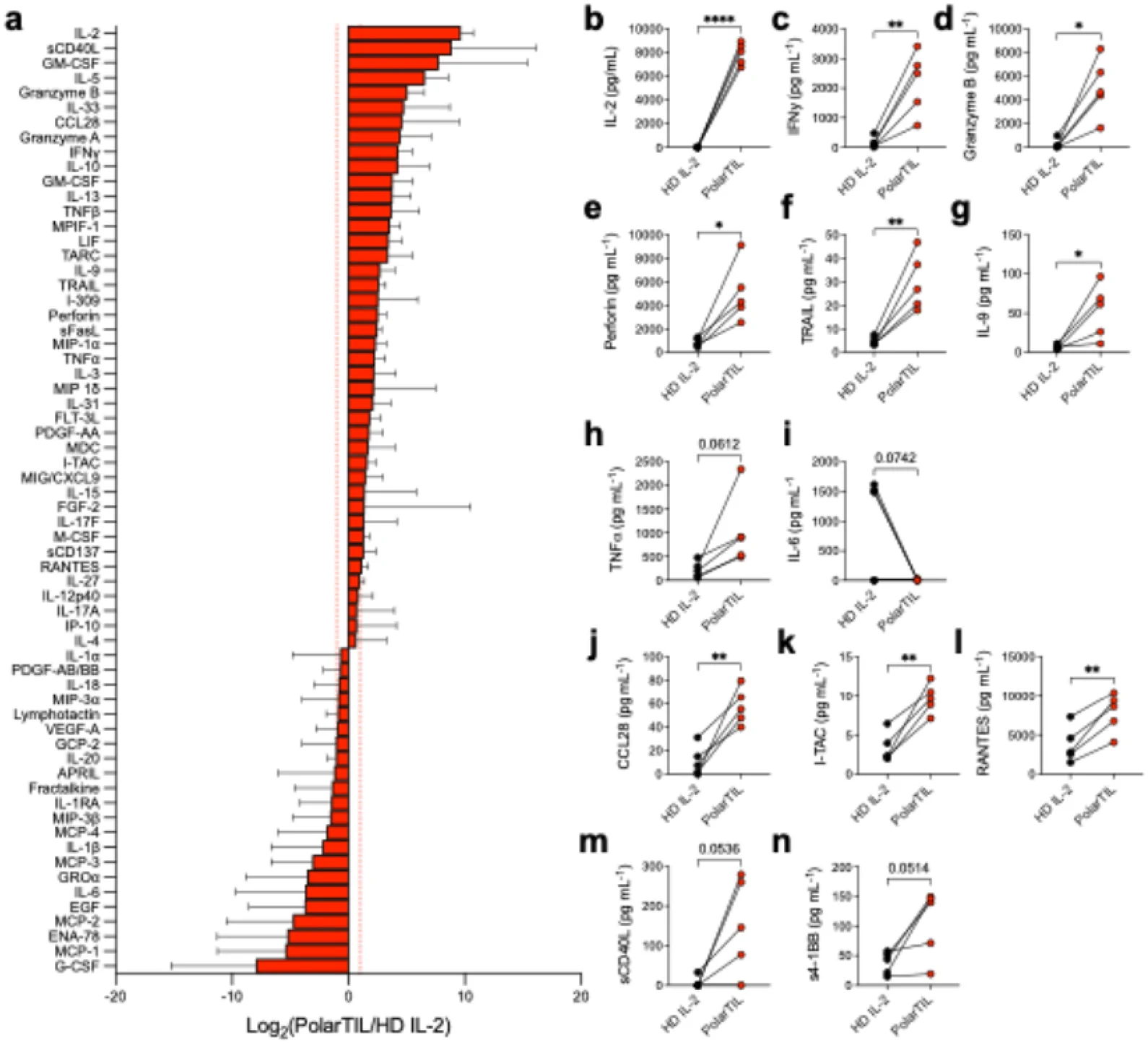
PolarTIL have an enhanced effector profile. **a**, Fold change (log_2_) of PolarTIL vs. HD-IL2 cytokine production as measured by 96-plex Luminex assay within cell culture supernatant from reactivated TIL cultures (day 3 post-reactivation with anti-CD3; n=5). Concentrations (pg/mL) of individual cytokines and chemokines of interest are reported per culture condition: IL-2 (**b**), IFNγ (**c**), Granzyme B (**d**), Perforin (**e**), TRAIL (**f**), IL-9 (**g**), TNF (**h**), IL-6 (**i**), CCL28 (**j**), I-TAC (**k**), RANTES (**l**), CD40L (**m**), and s4-1BB (**n**); n=5, paired T-test. * = p<0.05, ** = p<0.01, **** = p<0.0001.

**Figure 4:**
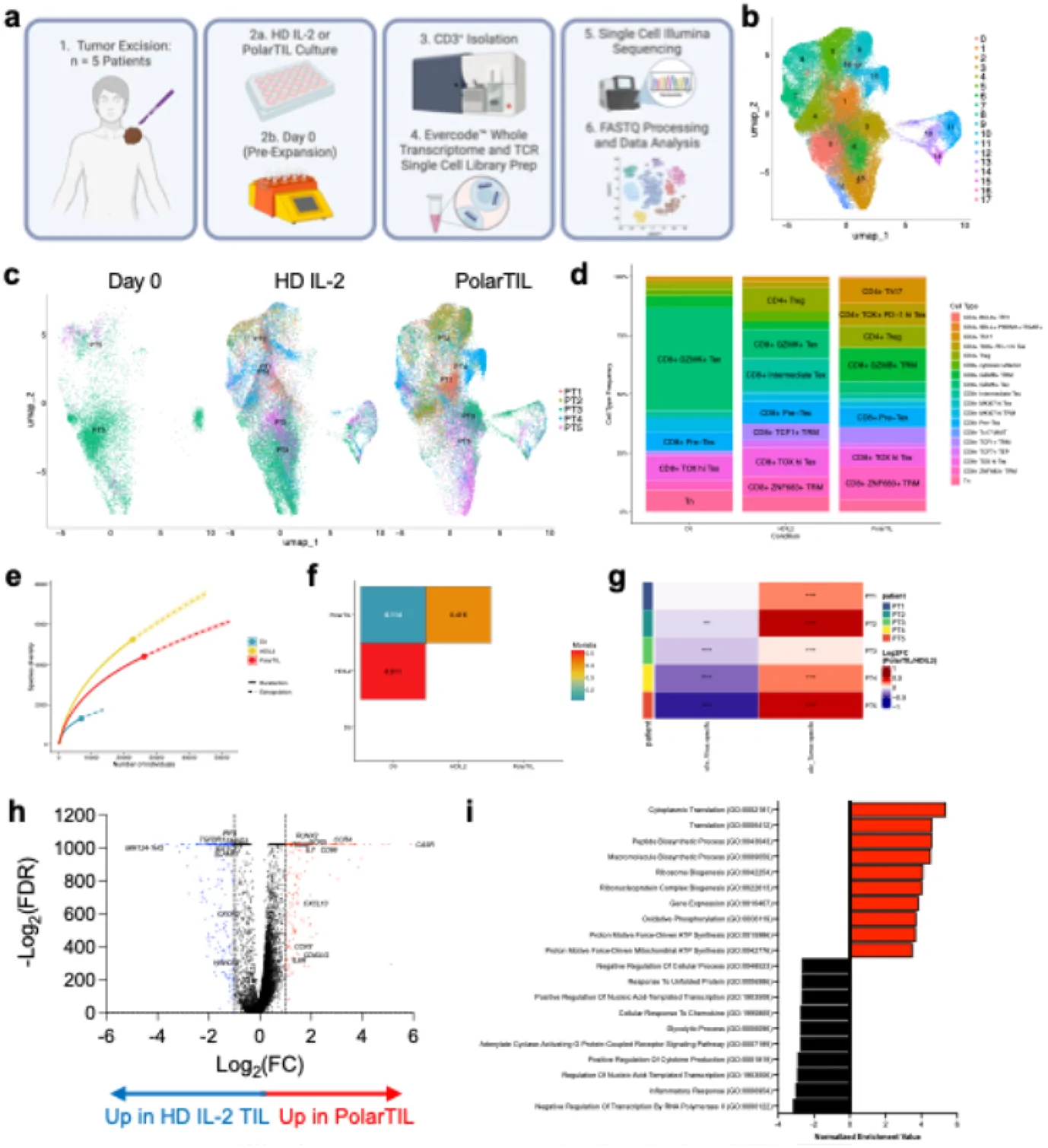
PolarTIL are transcriptionally distinct from HD IL-2 TIL. **a**, Experimental schematic. HD IL-2 TIL or PolarTIL were cultured from n=5 patients. The remainder of the tumor was digested into a single cell suspension and cryopreserved as matched “Day 0 Pre-expansion” TIL. Following expansion, TIL were cryopreserved until sequencing preparations. Following recovery from cryo, HD IL-2 TIL, PolarTIL and Day 0 TIL were FACS sorted to isolate pure CD3+ cells, which were then taken through the Evercode Whole Transcriptome and TCR single cell sequencing library preparation (Parse Biosciences). Library preparations were then QC’d and sequenced using Illumina sequencing technology. The resulting FASTQ files were processed with Parse’s pipeline and analyzed with R. **b**, Resulting clustered UMAP of pooled cells from all patients and samples. **c**, Distribution of cells from each patient within Day 0, HD IL-2 and PolarTIL culture conditions. **d**, Frequency of annotated cell types within the culture conditions. Labeled bars indicate cell types with >7% frequency. **e**, Sample-size-based TCR clonal rarefaction and extrapolation curve for each group (Shannon Diversity Index). **f**, TCR clonal overlap analysis between groups (Morisita Overlap Index). **g**, Heatmap depicting log2fold change (FC) in average virus- and tumor-reactivity gene signature scores per TCR clonotype for PolarTIL versus HD IL-2 TIL by patient (unpaired Wilcoxon signed-rank test). **h**, Volcano plot displaying Log2FC (x-axis) vs. −Log2False Discovery Rate (FDR) for PolarTIL vs. HD IL-2 TIL. Red data points indicate genes significantly upregulated in PolarTIL, while blue points are significantly upregulated in HD IL-2 TIL. Dashed lines are at -1 and 1 Log2FC. **i**, Gene ontology (GO) pathway analysis of PolarTIL vs. HD IL-2 TIL. Red bars are enriched in PolarTIL, black are enriched in HD IL-2 TIL. *** = p<0.001, **** = p<0.0001.

**Figure 5:**
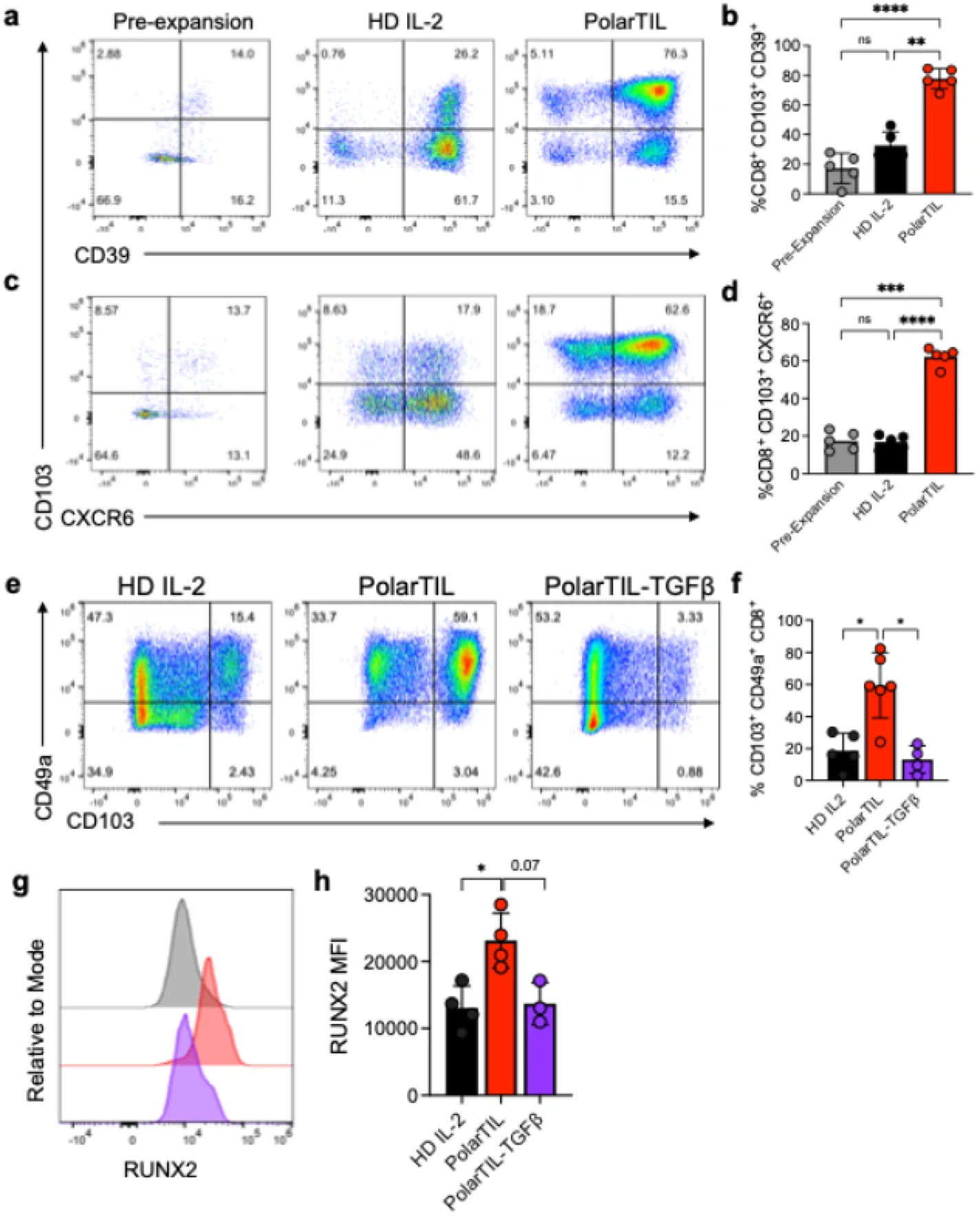
Enrichment of resident memory phenotype in PolarTIL is controlled by TGF-β1. **a**, Representative flow cytometry plots of CD39 (x-axis) and CD103 (y-axis) expression of CD8^+^ pre-expansion day 0 TIL, HD IL-2 TIL and PolarTIL. **b**, Average (±SD) percent of CD103^+^ CD39^+^ CD8^+^ T cells (n=5, one-way ANOVA with Šídák’s multiple comparisons test). **c**, Representative flow cytometry plots of CXCR6 (x-axis) and CD103 (y-axis) expression of CD8^+^ pre-expansion day 0 TIL, HD IL-2 TIL and PolarTIL. **d**, Average (±SD) percent of CD103^+^ CXCR6^+^ CD8^+^ T cells (n=5, one-way ANOVA with Šídák’s multiple comparisons test). **e**, Representative flow cytometry plots of CD103 (x-axis) and CD49a (y-axis) expression of CD8^+^ HD IL-2 TIL, PolarTIL, and PolarTIL-TGFβ. **f**, Average (±SD) percent of CD103^+^ CD49a^+^ CD8^+^ T cells (n=6, one-way ANOVA with Šídák’s multiple comparisons test). **g**, Representative flow cytometry of RUNX2 expression on CD8+ T cells in HD IL-2 TIL (grey), PolarTIL (red) and PolarTIL-TGFβ (purple). **h**, Average (±SD) MFI of RUNX2 expression on CD8+ TIL (n=3-4, mixed-effects analysis with Šídák’s multiple comparisons test). * = p<0.05, ** = p<0.01, *** = p<0.001, **** = p<0.0001.

**Figure 6:**
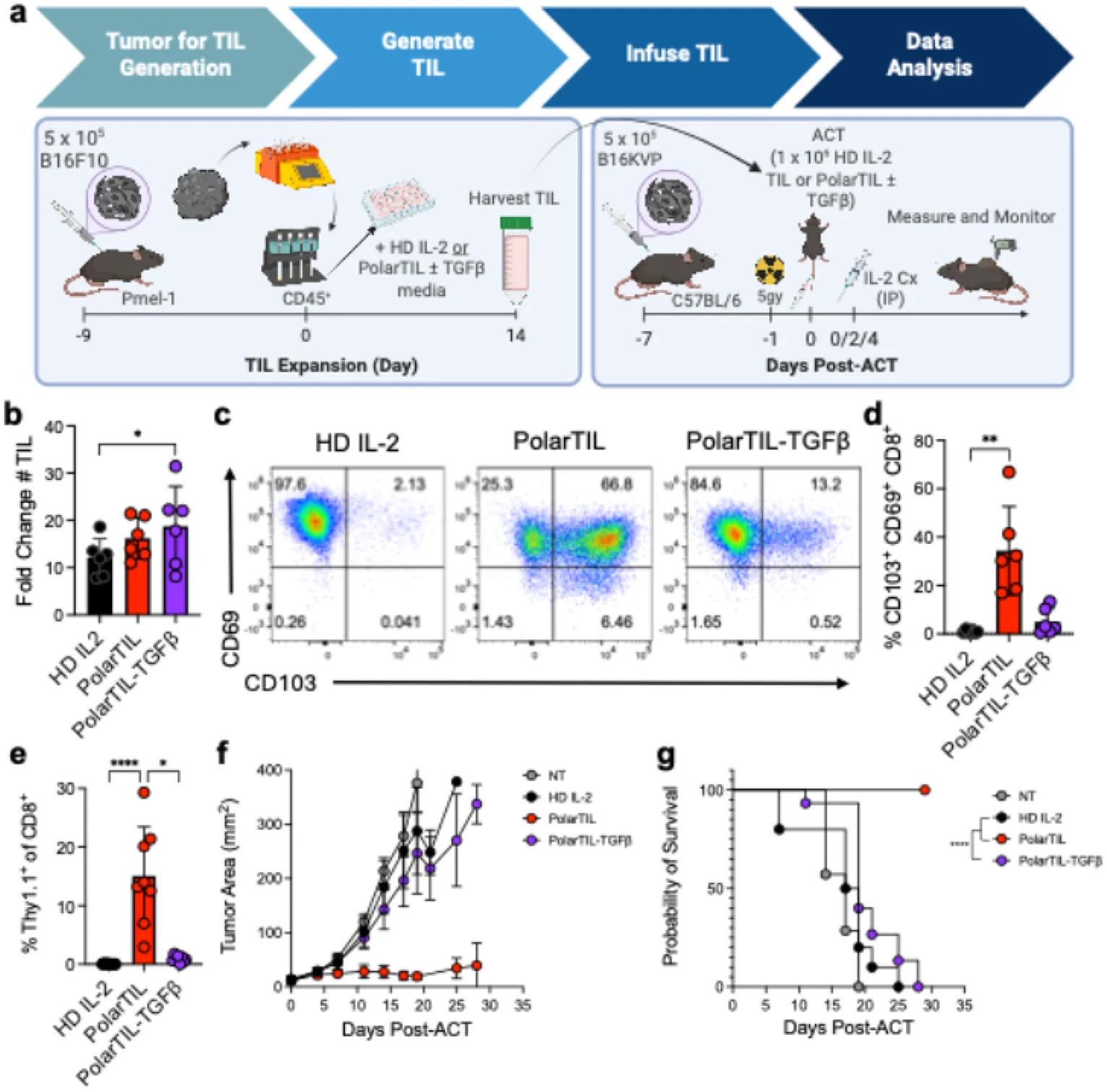
TGF-β1 is critical for PolarTIL performance. **a**, Murine TIL experiment schematic. CD45^+^ TIL were isolated from established B16F10 melanoma tumors harvested from Pmel-1 mice and cultured with media containing mCD3 and either HD IL-2 (6000 IU/mL IL-2) or PolarTIL cytokines (IL-1β, IL-6, IL-21, ±TGF-β1, 200IU/mL IL-2, mIL-4 and mIFNγ) for two weeks. Expanded TIL were adoptively transferred into lymphodepleted (5gy total body irradiation) C57BL/6J mice bearing established B16KVP tumors. TIL-therapy treated mice received IL-2 complex (rhIL-2 + IL-2) on days 0, 2, and 4 post-adoptive cell therapy (ACT). Mice were monitored for tumor area until endpoint criteria was met. **b**, Fold change in final TIL count at end of culture (n=6). **c**, Representative flow plots, CD103 (x-axis) vs CD69 (y-axis). Values are percent of CD8+ T cells. **d**, Average (± SD) percent CD103^+^ CD69^+^ of CD8^+^ T cells (n=6, one-way ANOVA with Šídák’s multiple comparisons test). **e**, Presence of donor Thy1.1^+^ TIL in peripheral blood of treated mice 11 days post-ACT (n=8/group, Kruskal-Wallis test with Dunn’s multiple comparisons test). **f**, Average tumor area (± SD) over time of no treatment control (gray, n=10), HD IL-2 TIL (black, n=10), PolarTIL (red, n=10), and PolarTIL-TGF-β1 (purple, n=15) treated mice. **g**, Probability of survival over time (Kaplan-Meier with Holm-Šídák’s multiple comparisons test). * = p<0.05, ** = p<0.01, **** = p<0.0001.

## Data Availability

The single cell data generated in this study are publicly available in Gene Expression Omnibus (GEO) submission GSE346066. All other raw data are available upon request from the corresponding author.
